# Supplementary material for: Intragenomic Variability of ITS Sequences in Bjerkandera adusta
Source: J Fungi (Basel). 2022 Jun 22;8(7):654. doi: 10.3390/jof8070654 (PMC9319410; doi:10.3390/jof8070654)
Supplement: Supplementary file 1 [file jof-08-00654-s001.zip › jof-1767609-supplementary.pdf]

Table S1: Origin of specimens and variability of *Bjerkandera adusta* ITS sequences obtained through this study.

Specimen	Origin and host				GenBank Accession numbers		Sequence polymorphism at position				Resulting ITS type
	Locality	Coordinates	Altitude m (a.s.l.)	Host	ITS	tef1-α	ITS1	ITS2			
							157	388	467-468	522	
913	Sala	N48.15056° E17.86953°	123	unknown	ON391760	XX	C	T	AG	G	A
692	Bratislava	N48.14658° E17.07328°	152	*Corylus avellana*	ON391761	ON411223	C	T	AG	G	A
B1	Muranska planina	N48.76664° E20.07542°	690	unknown	ON391762	XX	C	T	AG	G	A
738	Bratislava	N48.15902° E17.08899°	213	*Quercus* sp.	ON391763	XX	C	T	AG	G	A
766	Namestovo	N49.40535° E19.4795°	611	*Aesculus hippocastanum*	ON391764	ON411224	C	T	AG	G	A
1749	Presov	N49.00797° E21.23379°	224	*Salix babylonica*	ON391765	ON411228	C	T	AG	G	A
520	Brezno	N48.8076° E19.632°	500	*Betula pendula*	ON391766	XX	C	T	AG	G	A
676	Bratislava	N48.16291° E17.05386°	188	*Betula pendula*	ON391767	ON411229	T	C	GA	A	B
B2	Streda nad Bodrogom	N48.37054° E21.7512°	98	unknown	ON391768	XX	T	C	GA	A	B
1490	Senica	N48.67583° E17.36847°	209	*Acer negundo*	ON391769	ON411227	T	C	GA	A	B
859	Galanta	N48.19206° E17.73846°	119	unknown	ON391770	XX	Y	Y	RR	R	A x B
957	Piestany	N48.58861° E17.84443°	168	*Acer negundo*	ON391771	ON411226	Y	Y	RR	R	A x B
931	Surany	N48.0907° E18.18433°	124	unknown	ON391772	XX	Y	Y	RR	R	A x B
884	Sala	N48.14824° E17.86687°	116	*Aesculus hippocastanum*	ON391773	ON411225	Y	Y	RR	R	A x B
898	Sala	N48.14812° E17.86811°	117	unknown	ON391774	XX	Y	Y	RR	R	A x B
935	Surany	N48.08941° E18.18442°	125	unknown	ON391775	XX	Y	Y	RR	R	A x B

994	Zarnovica	N48.48095° E18.71931°	228	unknown	ON391776	XX	Y	Y	RR	R	A x B
902	Sala	N48.14852° E17.86614°	117	unknown	ON391777	XX	Y	Y	RR	R	A x B

Locality, GPS coordinates, altitude and host species (if available) are shown together with GenBank accession numbers for ITS and tef1-α sequences obtained through this study. Sequence at specific position of ITS sequences is shown together with final typization of isolates based on sequence polymorphisms.
